# Negative enthalpy alloys and local chemical ordering: a concept and route leading to synergy of strength and ductility

**DOI:** 10.1093/nsr/nwae026

**Published:** 2024-01-18

**Authors:** Zibing An, Tao Yang, Caijuan Shi, Shengcheng Mao, Lihua Wang, Ang Li, Wei Li, Xianmeng Xue, Ming Sun, Yifan Bai, Yapeng He, Fuzeng Ren, Zhouguang Lu, Ming Yan, Yang Ren, Chain-Tsuan Liu, Ze Zhang, Xiaodong Han

**Affiliations:** Beijing Key Lab of Microstructure and Property of Advanced Materials, College of Materials Science & Engineering, Beijing University of Technology, Beijing 100124, China; Department of Materials Science and Engineering, City University of Hong Kong, Hong Kong, China; Key Laboratory of Partial Acceleration Physics & Technology, Institute of High Energy Physics, Chinese Academy of Sciences, Beijing 100049, China; Beijing Key Lab of Microstructure and Property of Advanced Materials, College of Materials Science & Engineering, Beijing University of Technology, Beijing 100124, China; Beijing Key Lab of Microstructure and Property of Advanced Materials, College of Materials Science & Engineering, Beijing University of Technology, Beijing 100124, China; Beijing Key Lab of Microstructure and Property of Advanced Materials, College of Materials Science & Engineering, Beijing University of Technology, Beijing 100124, China; Beijing Key Lab of Microstructure and Property of Advanced Materials, College of Materials Science & Engineering, Beijing University of Technology, Beijing 100124, China; Beijing Key Lab of Microstructure and Property of Advanced Materials, College of Materials Science & Engineering, Beijing University of Technology, Beijing 100124, China; Beijing Key Lab of Microstructure and Property of Advanced Materials, College of Materials Science & Engineering, Beijing University of Technology, Beijing 100124, China; Beijing Key Lab of Microstructure and Property of Advanced Materials, College of Materials Science & Engineering, Beijing University of Technology, Beijing 100124, China; Beijing Key Lab of Microstructure and Property of Advanced Materials, College of Materials Science & Engineering, Beijing University of Technology, Beijing 100124, China; Department of Materials Science & Engineering, Southern University of Science and Technology, Shenzhen 518055, China; Department of Materials Science & Engineering, Southern University of Science and Technology, Shenzhen 518055, China; Department of Materials Science & Engineering, Southern University of Science and Technology, Shenzhen 518055, China; Department of Physics, City University of Hong Kong, Hong Kong, China; Department of Materials Science and Engineering, City University of Hong Kong, Hong Kong, China; Beijing Key Lab of Microstructure and Property of Advanced Materials, College of Materials Science & Engineering, Beijing University of Technology, Beijing 100124, China; State Key Laboratory of Silicon Materials, Department of Materials Science and Engineering, Zhejiang University, Hangzhou 310058, China; Beijing Key Lab of Microstructure and Property of Advanced Materials, College of Materials Science & Engineering, Beijing University of Technology, Beijing 100124, China; Department of Materials Science & Engineering, Southern University of Science and Technology, Shenzhen 518055, China

**Keywords:** negative enthalpy alloys, local chemical ordering, strength, ductility, dislocation

## Abstract

Solid solutions are ubiquitous in metals and alloys. Local chemical ordering (LCO) is a fundamental sub-nano/nanoscale process that occurs in many solid solutions and can be used as a microstructure to optimize strength and ductility. However, the formation of LCO has not been fully elucidated, let alone how to provide efficient routes for designing LCO to achieve synergistic effects on both superb strength and ductility. Herein, we propose the formation and control of LCO in negative enthalpy alloys. With engineering negative enthalpy in solid solutions, genetic LCO components are formed in negative enthalpy refractory high-entropy alloys (RHEAs). In contrast to conventional ‘trial-and-error’ approaches, the control of LCO by using engineering negative enthalpy in RHEAs is instructive and results in superior strength (1160 MPa) and uniform ductility (24.5%) under tension at ambient temperature, which are among the best reported so far. LCO can promote dislocation cross-slip, enhancing the interaction between dislocations and their accumulation at large tensile strains; sustainable strain hardening can thereby be attained to ensure high ductility of the alloy. This work paves the way for new research fields on negative enthalpy solid solutions and alloys for the synergy of strength and ductility as well as new functions.

## Teaser

Negative enthalpy alloys act as a new concept and route for introducing various degree of local chemical ordering heterostructures, leading to superb synergy of strength and ductility.

## INTRODUCTION

Solid solutions are ubiquitous crystalline structures that are widely used in metals and alloys. In solid solutions, local chemical ordering (LCO) is a fundamental sub-nano/nanoscale structure [1–8] and is described as the chemical order present in neighboring atomic shells and local domains (the definition is described in the ‘Methods’ section). LCO includes short-range ordering (SRO) on a small size scale (<1 nm, covering only the nearest and second nearest neighbor shells), medium-range ordering (MRO) (the size range of 1∼5 nm) and long-range ordering (LRO) nanodomains (dimensions of >5 nm) [1,9,10]. LCO is present in some face-centered cubic (FCC)-structured high/medium-entropy alloys (H/MEAs) [1,7,9]. Several body-centered cubic (BCC)-structured refractory high-entropy alloys (RHEAs) with LCO structures, such as NbMoTaW, Ti_50_Zr_18_Nb_15_V_12_Al_5_ and (Ti_38_V_15_Nb_23_Hf_24_)_95_Al_5_ alloys, were also investigated [10–12]. However, to date, how to control the formation of LCO structures in metals and alloys has not been determined. More importantly, engineering LCO to achieve superb synergy of strength and ductility has been a longstanding challenge.

**Figure 1.**
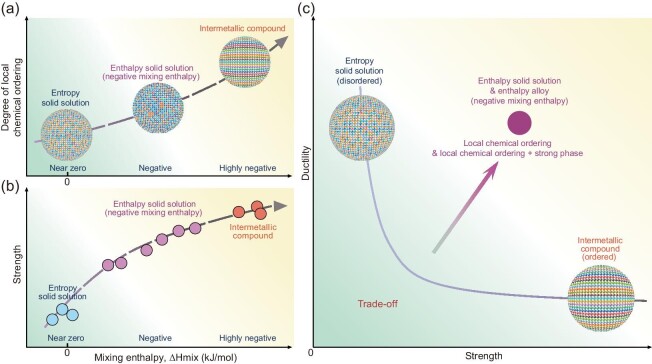
The ‘negative enthalpy solid solution/alloy’ concept and route toward synergy of high strength and ductility. Schematic sketches illustrating the relationships between (a) the degree of LCO and negative enthalpy, (b) the strength and negative enthalpy and (c) the synergy of strength and ductility. An entropy solid solution with near-zero enthalpy shows low strength but high ductility, while an intermetallic compound with highly negative mixing enthalpy exhibits mostly high strength but embrittlement, indicating a strength–ductility trade-off. Furthermore, negative enthalpy alloys with LCO heterostructures possess an unusual combination of strength and ductility, which are superior to those of entropy solid solutions and intermetallic compounds. Finally, through engineering a negative enthalpy, these LCO microstructures could be *in situ* composited with other strong nanophases, such as intermetallic compounds and metallic glass, to achieve higher strength in enthalpy alloys.

It was recently suggested that complex enthalpy interactions may occur at room temperature in HEAs [4]. The local chemical environment can be induced by low and even negative enthalpy [13,14] and large differences in electronegativity [15]. In addition, enthalpy-driven heterogeneities appear on the atomic scale to some degree [13,14]. Therefore, a negative enthalpy may be an indicator for the chemical affinity among atoms, derive atom clustering heterogeneities at different levels (e.g. chemical fluctuations and spinodal decomposition [16]) and cause LCO heterogeneities [4,13,14], thereby exhibiting enthalpy-related microstructure diversification. Inspired by these findings, we propose that, with negative enthalpy, an entropy solid solution could be broken and LCO could be introduced and engineered in solid solutions; i.e. the entropic solid solutions can be guided and directed by negative enthalpy to form a negative enthalpy solid solution [16] (the definition is described in the ‘Methods’ section) with LCO heterogeneities of different degrees until the intermetallic compounds are formed, as shown in Fig. 1a. Furthermore, with decreasing negative mixing enthalpy, the strength of an alloy can increase due to strong enthalpic interactions among the alloying atoms, which is termed negative mixing enthalpy strengthening [16], as shown in Fig. 1b. The high density of strong yet ductile LCO heterostructures spans multiple levels, allowing the alloys to block dislocations and instigate extensive intragranular dislocation sources, yielding large strain hardening. As a result, improvements in ductility and strength can be realized by varying the degree of LCO in negative enthalpy solid solutions and alloys (the definition is described in the ‘Methods’ section), as shown in Fig. 1c. More importantly, through engineering a negative enthalpy, these LCO microstructures could be *in situ* composited with other strong nanophases, such as intermetallic compounds and metallic glass, to achieve higher strength in negative enthalpy alloys.

To verify this hypothesis, a series of Hf–Nb–Ti–Ta–Al alloys with mixing enthalpies ranging from nearly zero to highly negative were produced in this research. To investigate the relationship between the mixing enthalpy and the degree of LCO heterogeneity, as well as the effect of the latter on the plastic deformation and mechanical properties of alloys, aberration-corrected scanning transmission electron microscopy (STEM) and energy-dispersive X-ray spectroscopy (EDS) in reciprocal and true spaces were employed. According to the results, an impressive yield strength (1160 MPa) along with the uniform tensile ductility (24.5%) was achieved in the HfNbTaTiAl_12_ RHEA. These findings not only provide atomistic insights into the LCO in RHEAs, but also furnish important information for engineering LCO to different degrees by tailoring the mixing enthalpy. Therefore, a negative enthalpy is proven to be essential for LCO design, providing superior mechanical properties at using far fewer resources compared with the traditional ‘trial-and-error’ methods.

## RESULTS AND DISCUSSION

### Microstructure and chemical distribution of HNTTA***_x_*** alloys

**Figure 2.**
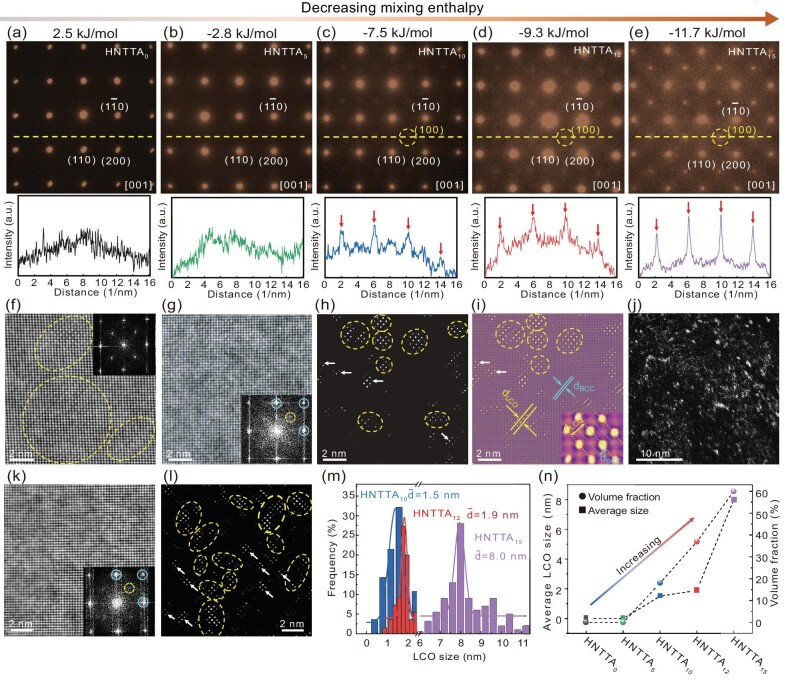
Microstructure of LCO in aged HNTTA*_x_* alloy. (a–e) SAED patterns under the [001] zone axis of the HNTTA_0_, HNTTA_5_, HNTTA_10_, HNTTA_12_ and HNTTA_15_ alloys. The diffraction data along the yellow dashed lines are plotted in the SAED images of the HNTTA_0_ (black), HNTTA_5_ (green), HNTTA_10_ (blue), HNTTA_12_ (red) and HNTTA_15_ (pink) alloys. (f) HAADF–STEM image of HNTTA_15_ alloy under [001] z.a. The inset is an FFT pattern. (g) HAADF–STEM image of HNTTA_10_ alloy under [001] z.a. The inset is an FFT pattern. Yellow circle: extra reflection. Blue circle: diffraction spot of the BCC phase. (h) Corresponding inverse FFT image revealing the LCO structure. (i) Corresponding IFFT image overlaid on the BCC lattice image. (j) Dark-field (DF)-TEM image of the HNTTA_10_ alloy showing bright entities in the LCO region. (k) HAADF–STEM image of the HNTTA_12_ alloy under the [001] z.a. Inset is the FFT pattern. (l) Corresponding IFFT image. (m) Size distribution of LCO determined by IFFT image of HNTTA_10_, HNTTA_12_ and HNTTA_15_ alloys. (n) Comparison of the LCO size and volume fraction in HNTTA*_x_* alloys.

Cast HfNbTiTaAl*_x_* (*x* = 0, 5, 10, 12 and 15) alloys were obtained by arc melting (hereafter referred to as HNTTA_0_, HNTTA_5_, HNTTA_10_, HNTTA_12_ and HNTTA_15_; additional details can be found in the Supplementary Data). The alloys exhibited a single-phase BCC structure consisting of dendritic and inter-dendritic regions, which indicated that elemental segregation occurred during the solidification process (Figs S1 and S2). After homogenization (1300°C for 2 h), cold rolling (70%) and aging (1100°C for 5 min and 400°C for 120 h), the dendrite and inter-dendrite structures disappeared (Fig. S3), indicating that the elements were homogeneously distributed. Scanning electron microscope‒EDS mapping also confirmed that the composition segregation was eliminated (Fig. S3). Table S1 displays the chemical analysis data, revealing the quasi-nominal composition of the alloys. Based on the rule of mixing, the mixing enthalpy ($\Delta {H}_{mix}$) was determined to be 2.5 kJ/mol (HNTTA_0_), –2.8 kJ/mol (HNTTA_5_), –7.5 kJ/mol (HNTTA_10_), –9.3 kJ/mol (HNTTA_12_) and –11.7 kJ/mol (HNTTA_15_) (see the Supplementary Data for more details). It is clear that, with the addition of Al, the mixing enthalpy of the alloys decreases, varying from positive to negative values, which is due to the more negative mixing enthalpy of the Al–R atom pairs compared with that of the R–R pairs (here, R = Hf, Nb, Ti or Ta) (Table S2). It has been demonstrated that the selected area electron diffraction (SAED) pattern along the varying zone axis (z.a.) can be one of the most fundamental pieces of evidence of LCO [3]. Figure 2a–c shows the SAED patterns of aged HNTTA_0_, HNTTA_5_ and HNTTA_10_ alloys along the [001] axis, respectively, which are fully indexed to the BCC structure. However, apart from a set of sharp diffraction spots from the BCC base, the extra superlattice reflections appear as many low-intensity spots along the [001] z.a. at the 1/2{200} positions of the BCC structure in the aged HNTTA_10_ alloy (indicated using yellow circles in Fig. 2c). Similarly, additional superlattice reflections are also observed along the [011] z.a. (Fig. S4). Meanwhile, no superlattice reflections are detected along the [111] z.a. (Fig. S4). Compared with those of the HNTTA_10_ alloy, the intensities of extra superlattice reflections of the 1/2{200} positions are enhanced in the HNTTA_12_ alloy (Fig. 2d), indicating an enhanced LCO structure. The high-intensity extra superlattice reflections of the 1/2{200} positions emerge in the aged HNTTA_15_ alloy, indicating the LRO B2 nanoprecipitate (Fig. 2e). Figure 2f shows the atomic-resolution high-angle annular dark-field scanning transmission electron microscopy (HAADF–STEM) image of HNTTA_15_ with the [001] z.a., revealing the size of the B2 nanoprecipitate. To demonstrate the diffuse extra discs more clearly, intensity line scans were performed along the yellow lines in the five SAEDs (see Fig. 2a–e), highlighting the weak extra peaks in the HNTTA_10_ alloy. Thus, coherent superlattice entities associated with lattice periodicity were detected in the BCC base of the alloys. At first glance, these coherent superlattices look like B2 structures. However, compared with those of the B2 phase (Fig. 2e), the intensities at the 1/2{200} positions are weak and exhibit no periodicity, indicating that the LRO B2 nanoprecipitate was free. It is thus speculated that the S/MRO structure exists in the HNTTA_10_ alloy.

Recent studies have suggested not only that LCO structures can introduce extra superlattice scattering, but also that planar defects (stacking faults) and higher-order Laue zone diffraction can introduce extra superlattice [17,18]. Therefore, to further confirm the LCO structure in the HNTTA_10_ and HNTTA_12_ alloys, atomic-resolution HAADF–STEM was employed. Figure 2g shows the HAADF–STEM image along the [001] z.a. of the HNTTA_10_ alloy, revealing the atomic lattice arrangement. In turn, the inset shows the fast Fourier transform (FFT) pattern. Analogous to the SAED data, the superlattice reflections appear at similar 1/2{200} positions, one of which is highlighted using a yellow circle. Figure 2h displays the corresponding inverse FFT image obtained using extra reflections (1/2{200} spots). It is clear that the sizes of most superlattice regions (LCOs) fall within a range of 0.5–3 nm, as denoted using yellow circles. The dimensions of some LCOs are <1 nm, which can indicate the existence of four or six atoms (shown using white arrows). According to Wu *et al.*, the chemical SRO scale is <1 nm, while the chemical MRO extends from 1 to 5 nm [1,9]. Thus, both SRO and MRO coexist in the HNTTA_10_ alloy. Figure 2i displays the inverse FFT image overlaying the BCC lattice image. As seen from the magnified image in the inset, the spacings between the {200} planes in the LCO (*d_LCO_*, yellow lines) are twice that of the BCC lattice (*d_BCC_*, blue lines), which is consistent with the extra reflections appearing at the 1/2{200} positions in the diffraction pattern (Fig. 2c). Figure 2j shows a dark-field (DF) transmission electron microscopy (TEM) image of the HNTTA_10_ alloy obtained by using the additional spots in Fig. 2c, revealing the LCO structure (the light region). Similarly, the HAADF–STEM image along the [001] z.a. (Fig. 2k) and the inverse FFT image constructed on the basis of the extra reflections (1/2{200} spots) (Fig. 2l) reveal the SRO and MRO structures in the HNTTA_12_ alloy. Moreover, a few B2 nanoprecipitates with a size of ∼6 nm can be observed, indicating the presence of a complex of LCO and nanoprecipitate microstructures in the HNTTA_12_ alloy. The statistical size distribution within the LCO was determined based on the inverse FFT images, as shown in Fig. 2m. The average sizes ($\bar{d}$) of the LCOs were 1.5, 1.9 and 8.0 nm for the HNTTA_10_, HNTTA_12_ and HNTTA_15_ alloys, respectively, and the corresponding volume fractions were 17%, 36% and 59%, respectively (Fig. 2n).

Compared with the HNTTA_0_ and HNTTA_5_ alloys, which feature an identical state with different mixing enthalpies, the HNTTA_10_ and HNTTA_12_ alloys exhibit an LCO structure, indicating that negative enthalpy plays an important role in LCO formation. Moreover, compared with those in the HNTTA_10_ alloy, the size and volume fraction of the LCO in the HNTTA_12_ alloy increased, demonstrating that the LCO structure can be enhanced with decreasing mixing enthalpy (Fig. 2m and n). With a further decrease in the mixing enthalpy, a high-volume fraction of B2 nanoprecipitates formed in the HNTTA_15_ alloy. Therefore, with the route of negative enthalpy alloys design, LCOs can evolve to various degrees, and even nanoprecipitates, which can possess distinctive microstructures and mechanical properties as presented below.

**Figure 3.**
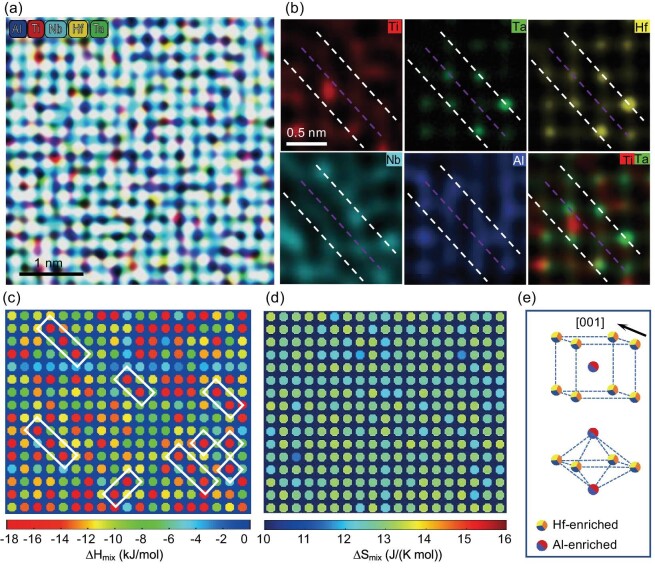
Chemical distribution in aged HNTTA_12_ alloy. (a) HAADF–EDS map. (b) Magnified HAADF–EDS maps of Hf, Ta, Ti, Ta, Al and Ti–Ta, respectively. (c) Atomic-level distribution of mixing enthalpy. (d) Atomic-level distribution of mixing entropy. (e) 3D unit cell of the LCO with a BCC lattice.

The chemical distribution of LCOs in the HNTTA_12_ alloy was investigated via EDS mapping using an UltraX detector as part of a TEM installation. In particular, specific arrangements of five chemical species, i.e. Hf, Nb, Ti, Ta and Al, were probed. Figure 3a displays the spatial distribution of each individual element from column to column. The brightness/darkness intensity of a column is closely related to the content of the examined element. In turn, Fig. 3b shows magnified EDS maps. Usually, the LCO can be well described in terms of Hf, Nb and Ta occupancy. As seen from the EDS maps, two (Hf, Nb, Ta)-enriched {200} planes could have formed a sandwich structure with one (Hf, Nb, Ta)-depleted {200} plane (see the map for Hf, Nb and Ta, under dashed white lines). Arranging the maps in another way reveals that the (Hf, Nb, Ta)-enriched (200) planes could have alternated with those enriched in Ti and Al (e.g. in the Ti–Ta map, the dashed pink line crosses the bright red spots but with a faint or even vanishing green Ta background). Figure 3c shows the mixing enthalpy distribution based on the atomically resolved chemical maps, which exhibit substantial fluctuations. More importantly, a sandwich structure formed by two {200} planes with a slightly negative mixing enthalpy (blue) and one {200} plane with a highly negative mixing enthalpy (red) can be observed (highlighted using a white box), further confirming the important role of mixing enthalpy in the formation of LCO structures. In contrast, the mixing entropy distribution appears uniform (Fig. 3d). Figure 3e depicts a 3D unit cell of LCO with a BCC lattice. Since the two Hf/Ta/Nb-enriched planes are separated by a distance that is twice the normal spacing of the {200} planes in the BCC lattice, there is one Ti/Al-enriched plane in between. Therefore, the extra superlattice scattering appeared at the 1/2{200} position (Fig. 2d) and extinction was observed along the [111] z.a. (Fig. S4).

### Formation mechanism for the LCO structure

It is clear that, with decreasing enthalpy, the LCO structure is enhanced, exhibiting microstructural transformation from an entropy solid solution to an LCO heterostructure or even to B2 nanoprecipitates, which agrees well with the images in Fig. 1a. These results establish a relationship between the mixing enthalpy and degree of LCO formation, where a decrease in the mixing enthalpy causes an increase in the degree of LCO formation. According to the EDS maps, the {200} planes in the BCC and the 1/2{200} planes in the LCOs were alternatively enriched with the (Hf, Nb, Ta) and (Al, Ti) components (Fig. 3). Given the more negative mixing enthalpy values of the Hf–Al (–39 kJ/mol), Ta–Al (–19 kJ/mol) and Nb–Al (–18 kJ/mol) pairs (Table S2), it can be inferred that these pairs show the great tendency to bond, exhibiting an ordered atomic arrangement and thereby becoming the first-order nearest neighbors. In contrast, the R–R atom pairs with near-zero mixing enthalpies tend to avoid each other. Thus, the R-rich atomic columns with near-zero mixing enthalpies are in close proximity to the Al-rich columns with negative mixing enthalpies, thereby forming a sandwich structure (Fig. 3c). Wu *et al.* [1,4,13,14,19] and Ma *et al.* [4,20] have suggested that the enthalpy interaction can be a key indicator of the chemical affinity among atoms, indicating the propensity for atom clustering and the formation of LCO. Therefore, for the HNTTA_0_ (2.5 kJ/mol) and HNTTA_5_ (–2.8 kJ/mol) alloys, which have mixing enthalpies close to zero, an entropy solid solution with a disordered BCC structure was obtained (Fig. S4). To further confirm this trend, another alloy with a HNTTA_18_ composition was produced. The mixing enthalpy of the HNTTA_18_ alloy is –14.0 kJ/mol. Based on the microstructure analysis, the HNTTA_18_ alloy possesses a global B2 structure (Fig. S5). This result highlights the effect of mixing enthalpy on the degree of ordering: the lower the mixing enthalpy, the greater the degree of ordering (Fig. 1a). To exclude the mixing entropy effect, a series of (HfNbTiTa)_100__–_*_x_*V*_x_* alloys (hereafter referred to as HNTTV*_x_; x* = 5, 10, 12) were fabricated and their mixing entropy was similar to that of HNTTA*_x_* alloys. The mixing enthalpy values of the HNTTV_5_, HNTTV_10_ and HNTTV_12_ alloys were found to be 2.0, 1.5 and 1.3 kJ/mol, respectively, all approaching zero. According to the TEM analysis, the HNTTV_5_, HNTTV_10_ and HNTTV_12_ alloys had an entropy solid solution structure similar to that of the HNTTA_0_ alloy (Fig. S6). Thus, it can be concluded that the entropy plays a small role in the formation of LCO, which coincides with the images in Fig. 3d. Identical results were also observed for the FCC alloys. As a representative example of an alloy whose mixing enthalpy is close to zero, the CoNiFe alloy possesses a mixing enthalpy of –1.3 kJ/mol, indicating an entropy solid solution structure [21]. In contrast, CoCrNi alloys with a mixing enthalpy of –4.9 kJ/mol exhibit an LCO heterostructure, indicating an enthalpy solid solution structure [4]. Based on these data, it can be inferred that the negative enthalpy values serve as the ‘genetic’ feature of an alloy, determining whether the LCO structure will form. Hence, the LCO structure can be engineered by decreasing the enthalpy of the system, promoting the transformation from entropy solid solution/alloys to enthalpy solid solution/alloys, which is called the negative enthalpy solid solution/alloy design strategy.

### Tensile properties of HNTTA***_x_*** alloys

**Figure 4.**
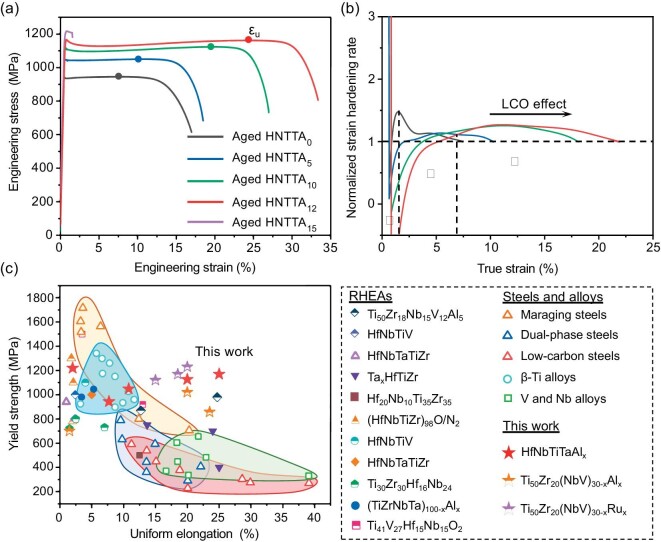
Tensile properties of aged HNTTA*_x_* alloys. (a) Engineering stress–strain curves. (b) Corresponding normalized strain-hardening rate versus true strain curves and true stress–strain curves. (c) The yield strength versus uniform elongation map of the HNTTA*_x_* alloy in comparison with other BCC alloys, including BCC RHEAs [10,23–32]; maraging, dual-phase steels and low-carbon steels; β-Ti alloys, V alloys and Nb alloys.

Figure 4a displays the engineering stress–strain curves of aged HNTTA*_x_* alloys. With decreasing mixing enthalpy, the yield strength (*σ_y_*) increased from 943 MPa for the HNTTA_0_ alloy to 1220 MPa for the HNTTA_15_ alloy. More importantly, the appearance and enhancement of LCO can improve the uniform tensile ductility (*ε_u_*) until a high-volume fraction of brittle B2 nanoprecipitates is formed. The ductility of the HNTTA_12_ alloy reached 24.5%, which is 256% that of the NHTTA_0_ alloy. Thus, the HNTTA_12_ alloy exhibited the best combination of yield strength and ductility.

For metals and alloys, high strain hardening is a primary reason for preventing necking so as to reach high ductility. According to the Considère criterion, when the strain-hardening rate is lower than the true stress, there is the plastic flow localization, leading to necking [22]. Moreover, the normalized strain-hardening rate can be calculated as [23]:


(1) \begin{eqnarray*}
\theta = \frac{{\partial {\sigma }_{\scriptsize\textit{true}}}}{{\partial {\varepsilon }_{\scriptsize\textit{true}}}} \cdot \frac{1}{{{\sigma }_{\scriptsize\textit{true}}}},
\end{eqnarray*}

where ${\sigma }_{\scriptsize\textit{true}}$ is the true stress, ${\varepsilon }_{\scriptsize\textit{true}}$ is the true strain and $\frac{{\partial {\sigma }_{\scriptsize\textit{true}}}}{{\partial {\varepsilon }_{\scriptsize\textit{true}}}}$ is the strain-hardening rate. Therefore, the emergence of necking in a system is determined by the criterion of $\theta < 1$. Figure 4b displays the normalized strain-hardening rates of the alloys as functions of true strain to clearly show the relationship between the strain-hardening rate and true stress, and thereby determining whether necking occurs. At the early deformation stages (Ⅰ, Ⅱ), there is a decrease in the $\theta $ value followed by a slow increase for the alloy with a more negative mixing enthalpy. At the later deformation stage (Ⅲ), a steadily high $\theta $ value (>1) was observed for the HNTTA_12_ alloy, resulting in an increase in the uniform tensile ductility. This reveals the enhancing effect of LCO on strain hardening, especially in the late deformation period. Figure 4c shows the tensile properties (*σ_y_* and *ε_u_*) of HNTTA*_x_* alloys and other BCC structure alloys (e.g. RHEAs, high-strength steels (maraging, dual-phase and low-carbon steels), β-Ti alloys and V/Nb alloys) [10,24–33]. It is clear that the HNTTA_12_ alloy outperforms most of those alloys due to its unusual strength−ductility combination.

### The effect of the LCO structure on the strain hardening and deformation mechanism

Before discussing the strain hardening and deformation mechanism, the increase in yield strength among the five alloys is explained, as shown in Fig. 4a. The reason is that the stronger bonding between atoms with a more negative enthalpy contributes to the increase in lattice friction, thereby increasing the yield strength. There is a quasilinear relationship between the yield strength and mixing enthalpy of the alloys, i.e. $\Delta {\sigma }_y = {\sigma }_0 + K\Delta {H}_{mix}$ (Fig. 1b). The negative mixing enthalpy strengthening coefficient ($| K |$) is determined to be ∼32 MPa/(kJ/mol), which is consistent with the results of previous studies (e.g. Zr_1.2_V_0.8_NbTi_3.6_Al*_x_* (∼27 MPa/(kJ/mol)) [34], Ti_3_Zr_1.5_NbVAl*_x_* (∼28 MPa/(kJ/mol)) [35], (NbTiZr)_100−_*_x_*Al*_x_* (∼27 MPa/(kJ/mol)) [36] and (TiZrHfNb)_100−_*_x_*Al*_x_* (∼28 MPa/(kJ/mol)) [37].

**Figure 5.**
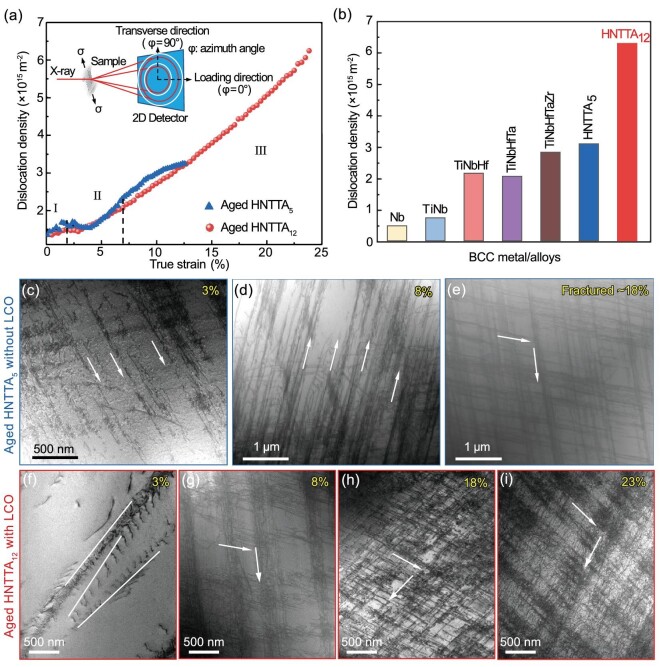
Dislocation density and deformation mechanisms of the aged HNTTA_5_ and aged HNTTA_12_ alloys at room temperature. (a) Dislocation density of HNTTA_5_ and HNTTA_12_ alloys as a function of the tensile true strain. Inset shows a schematic of *in situ* XRD tests. (b) The limited dislocation density of HNTTA_12_ alloy in comparison with other BCC metals and alloys. (c) TEM image of the HNTTA_5_ alloy at tensile strain of 3% showing dislocation bands structure (white arrows). (d) TEM image of the HNTTA_5_ alloy at tensile strain of 8%. (e) TEM image of the HNTTA_5_ alloy after tensile fracture at stain of ∼18% showing interacted dislocation bands. (f) TEM image of the HNTTA_12_ alloy at tensile strain of 3% showing dislocation cross-slip bands (white lines). (g) TEM image of the HNTTA_12_ alloy at tensile strain of 8% showing interacted dislocation bands. (h) TEM image of the HNTTA_12_ alloy at tensile strain of 18%. (i) TEM image of the HNTTA_12_ alloy at tensile stain of ∼23%.

Recall that a steadily high strain-hardening rate has earlier been observed in the HNTTA_12_ alloy (Fig. 4b). In general, strain hardening represents the resistance of a material to the dislocation motion so as to accommodate the applied strain [38,39]. Thus, the increased crystal defects, such as dislocations, twins, stacking faults and grain/phase boundaries, can inspire the strain hardening [14]. To the best of our knowledge, only dislocations are able to carry out the plastic deformation within BCC HEAs [30,34], which can be further confirmed via *in situ* synchrotron X-ray diffraction (XRD) tests. As shown in Fig. S7, during deformation, there were no diffraction peaks other than the BCC phase, indicating the lack of phase transformations. Therefore, the increase in dislocation density plays an important role in strain hardening, as demonstrated by Taylor [40–42]. Based on the Taylor equation, the strain-hardening rate can be estimated as:


(2) \begin{eqnarray*}
\frac{{\partial {\sigma }_{\scriptsize\textit{true}}}}{{\partial {\varepsilon }_{\scriptsize\textit{true}}}} = \textit{aMGb}\frac{{\partial \sqrt \rho }}{{\partial {\varepsilon }_{\scriptsize\textit{true}}}},
\end{eqnarray*}

where *a* is a constant, *M* is the Taylor factor, *G* is the shear modulus, *b* is the Burgers vector of dislocations and $\rho $ is the density of dislocations. Hence, the strain-hardening rate is dependent on the dislocation density during plastic deformation. Figure 5a displays the dislocation variation along the increased strain within the aged HNTTA_5_ (without LCO) and HNTTA_12_ (with LCO) alloys, which was evaluated via *in situ* synchrotron XRD tests (the corresponding schematic is shown in the inset). At the first deformation stage Ⅰ, the dislocation density in HNTTA_12_ slowly increased from 1.3 × 10^15^ to 1.55 × 10^15^ m^−2^, which did not compensate for the increased flow stress, resulting in a decrease in the strain-hardening rate according to Equation (2). This can clearly explain the decrease in $\theta $ observed in Fig. 4b. A similar trend was observed in the HNTTA_5_ alloy. Subsequently, at the deformation stage Ⅱ, the HNTTA_5_ alloy showed a rapid increase in dislocation density, yielding a high strain-hardening rate, which coincided with a dramatic increase in $\theta $ (Fig. 4b). By contrast, the dislocation density and growth rate of the HNTTA_12_ alloy were lower than those of the HNTTA_5_ alloy, which resulted in a slower increase in $\theta $. At the large tensile strain (stage Ⅲ), the HNTTA_12_ alloy exhibited a sustainable increase in dislocation density at a rate greater than that of the HNTTA_5_ alloy, which reached 6.13 × 10^15^ m^−2^ at true strain of 23%. Figure 5b shows the limited dislocation densities of the HNTTA_12_ alloy and other BCC metals and alloys [43,44]. It is clear that the HNTTA_12_ alloy possesses a high density of dislocations, which ensures its high strain-hardening rate and $\theta $ value (Fig. 4b).

Therefore, it can be concluded that the LCO can affect the dislocation accumulation during the plastic deformation. Specifically, at the early deformation stage, the LCO delays the dislocation accumulation, while facilitating the sustainable dislocation activation and accumulation during the later deformation stage, which results in a higher density and steadily high strain-hardening rate and $\theta $ values.

To put the above mechanism into action, the TEM snapshots of samples at different tensile strains were further analysed, confirming the mechanical property data in Fig. 4a and b. The TEM images in Fig. 5c–i reveal the typical dislocation morphologies in HNTTA_5_ and HNTTA_12_ alloys at different interrupted plastic deformation strains (3%, 8%, 18% and 23%). At the early stage of deformation (3%), dislocation slip bands were observed in both alloys (Fig. 5c and f). However, compared with the HNTTA_12_ alloy, the dislocation bands in the HNTTA_5_ alloy demonstrated a small spacing, indicating a high density of dislocations, which matched well with Fig. 5a. Moreover, the dislocation cross-slip can be seen in the HNTTA_12_ alloy in the form of waved dislocation bands (white lines). Thus, it is speculated that the LCO can promote dislocation cross-slip. This trend has earlier been observed in other HEAs with the LCO structure [1,10]. This dislocation cross-slip reduces the dislocation density in the dislocation bands, releasing the local strain and yielding plastic deformation, which reduces strain-hardening ability [45] (Fig. 4b). However, at the later deformation strain stage (8%), the dislocation morphologies of the two alloys were significantly different (Fig. 5d and g). For the HNTTA_5_ alloy, the dislocation motion followed a planar slip way, forming multiple dislocation bands (Fig. 5d). By contrast, the second dislocation bands emerged in the HNTTA_12_ alloy to accommodate the plastic deformation. This promoted the dislocation interaction in these two slip bands, enhancing the strain-hardening rate, which was conducive to the increase in $\theta $ of the HNTTA_12_ alloy at the tensile stain of 8% (Fig. 4b). With a further increase in tensile strain (18%), the dislocation networks were detected in both the HNTTA_5_ and HNTTA_12_ alloys (Figs. 5e and h). Obviously, the HNTTA_12_ alloy showed a higher density of dislocations than the HNTTA_5_ alloy, which was consistent with the *in situ* XRD data. This observation also suggested the sustainable high strain-hardening capability of the HNTTA_12_ alloy. Figure 5i shows a TEM image of the HNTTA_12_ alloy at the tensile strain of 23%. In addition to the pre-existing planar slip bands, there are numerous dislocation tangles in the channels between slip bands, indicating cross-slip events inside the planar slip bands. Moreover, the dislocation density further increased, reaching a hitherto unreported high level (Fig. 5b). By contrast, this dislocation network-like structure was observed in the HNTTA_5_ alloy (Fig. 5e), although free of dislocations inside the region of the dislocation bands interaction.

**Figure 6.**
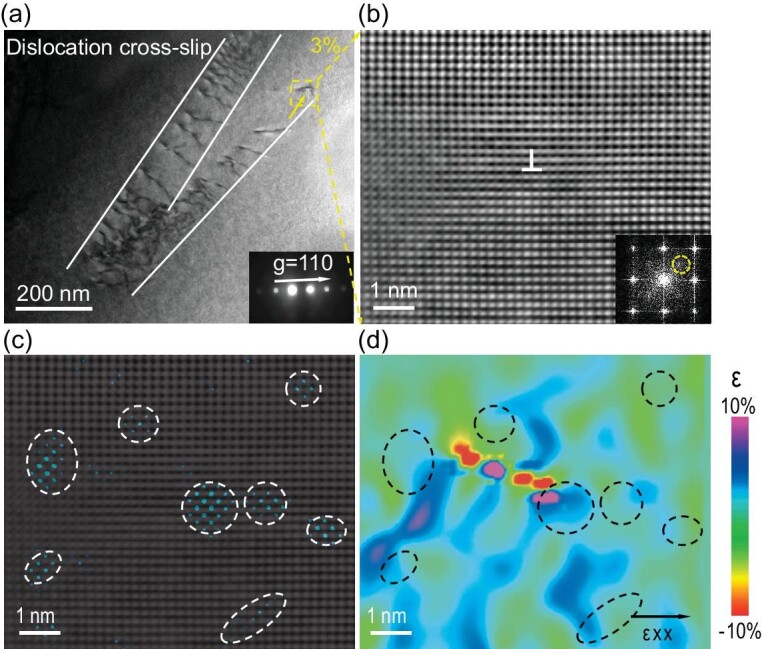
Mechanism of dislocation interacted with LCO. (a) TEM image of HNTTA_12_ alloy interrupted at 3% tensile strain showing dislocation cross-slip. (b) Atomic-resolution HAADF–STEM image of the local atomic structure of the region at the dislocation pinning point (yellow square). (c) Corresponding IFFT image overlaid on the BCC lattice image, revealing LCO structure. (d) Corresponding lattice strain mapping.

To understand the atomistic mechanisms of LCO-induced dislocation cross-slip in the HNTTA_12_ alloy, the HAADF–STEM snapshots of the dislocation bands were analysed. Figure 6a displays the dislocation dipoles produced by dislocation cross-slip. Moreover, the dislocation pining effect can be clearly observed (denoted using a yellow arrow), showing a curved dislocation line. Figure 6b depicts the magnified atomic-resolution HAADF–STEM image, corresponding to the yellow-highlighted region in Fig. 6a, which revealed the edge dislocations. It was thus evident that the curly dislocations possessed the mixed characteristics (Fig. 6a). Figure 6c contains the corresponding inverse FFT image overlaid with a BCC lattice image through the extra reflection 1/2{200} spots (see the inset in Fig. 6b). It is clear that lots of LCO structures have surrounded the edge dislocations, indicating the pining effect of LCO. The LCO drag dislocations and curved dislocation lines were also reported in the FCC-structured CoCrNi medium-entropy alloy [4]. Through the combination of the LCO and edge dislocations, a high lattice strain field formed *in situ* inside the dislocation bands (Fig. 6d), which interacted with the following dislocations. In turn, the high lattice strain field weakened the dislocation motion, which not only increased the dislocation interactions, but also enhanced the flow stress via improving the local stress field. As a result, the ‘dislocation cross-slip’ was improved (Figs 5f and 6a), thereby yielding two benefits. At first, it induced the plastic deformation. Second, it slowed down the ‘dislocation planar slip’ effects that conventionally occur in alloys with a single-phase structure, but also increased the dislocation density, which was in line with Fig. 5a and f–i.

Based on the above analysis, it can be inferred that the LCO structure facilitates the dislocation cross-slip via the pining dislocation movement, which produces two different effects at various tensile strain stages. At the beginning of plastic deformation, the dislocation cross-slip reduces the strain-hardening capability due to the lack of dislocation interaction. On the other hand, the dislocation cross-slip at the large tensile strain can promote the dislocation interaction with pre-existing slip bands, thus enhancing the dislocation density and strain hardening.

As a final note, to validate the wide applicability and confirm the high repeatability of negative enthalpy designing LCO features, the microstructure and mechanical properties of Ti–Zr–V–Nb–Al/Ru alloys were investigated as well (see the Supplementary Data). In these alloys, apart from the Al element, the mixing enthalpies of Ru–*X* (*X* = Ti, Zr, V, Nb) atom pairs are negative (Table S2); thus, the mixing enthalpy of the alloy can be tailored in a wide range from nearly zero to highly negative values. As a result, with the decrease in negative enthalpy, the LCO structures formed in the aged Ti_50_Zr_20_(VNb)_20_Al_10_ and Ti_50_Zr_20_(VNb)_15_Ru_5_ alloys (Figs S8–S13), indicating that negative enthalpy is the intrinsic driving force for formation LCO. In addition, with the development of LCO, the tensile strength and ductility of the alloys are simultaneously improved, showing a remarkable combination to overcome the strength–ductility trade-off shown in Fig. 4c (indicated using half-filled stars).

In summary, our results demonstrate that a negative enthalpy is essential for achieving LCO in RHEAs. Among the distinctive chemical elements, LCOs are formed in solid solutions and can be used as genetic characteristics for designing alloys. Putting this in a more general perspective, the negative enthalpy solid solution leads to the formation of genetic LCO, in which different degrees of LCO can be realized in solid solutions. Multilevel LCO heterogeneities break the randomness of the entropy solid solution structure, leading to the formation of chemical-affinity-biased negative enthalpy solid solutions and negative enthalpy alloys. The entropy solid solutions can be directed and evolve into negative enthalpy solid solutions and further to negative enthalpy alloys by negative enthalpy engineering of both the microstructures and mechanical properties. During plastic deformation, the LCO hinders dislocation sliding and promotes dislocation cross-slip, which not only induce deformation, but also facilitate dislocation interactions and improve the dislocation density at large strains, thereby enhancing the strain-hardening capability. As a result, the aged HNTTA_12_ negative enthalpy alloy with the enhanced LCO and nanoprecipitate heterostructures exhibited a superb synergy of the yield strength (1160 MPa) and uniform tensile ductility (24.5%). This strategy is readily expandable to other HEAs. Finally, LCO can potentially generate localized electronic states; thus, LCO can be used to design versatile functional materials in addition to providing synergistic effects on material strength and ductility.

## METHODS

### Definition of LCO

LCO refers to structures that appear in a solid solution containing more than two constituent elements [1–8]. LCO is normally formed by biased chemical interactions among distinctive atoms with negative mixing enthalpy values in solid solutions. LCO describes the chemical order present in neighboring atomic shells and local domains, including sub-nanoscale SRO structures, nanoscale MRO structures and nanoscale LRO domains in a solid solution, stemming from negative mixing enthalpic interactions [1,9]. Usually, the size of the SRO is <1 nm, which is only the nearest and second nearest neighbor shells. The MRO is a larger LCO than the SRO but is still much smaller than traditional LRO nanoprecipitates. The size of the MRO was temporarily defined as 1∼5 nm. Noticeably, LCO can extend the long-range nanometer scale, but it is not long-range chemical-ordered in all three dimensions to the spatial extent or the near-perfect degree of chemical order (nanoprecipitates). Chemical ordering of LCO only partially develops within a limited spatial extent, although it may occur over a long range in another dimension. Thus, LCO can be viewed only in specific directions in the experiments. Moreover, the extra diffraction signal of LCO is weak and diffuse in the SAED patterns. That is to say, in the view of crystal periodicity, LCO is expected to be an indispensable intermediate link between ideal disordering and the final stable LRO of intermetallic compounds.

### Definition of the entropy solid solution and negative enthalpy solid solution

Entropy solid solution is used to describe a disordered solid solution structure in which the mixing enthalpy (mixing heat) among the distinctive alloying element atoms is near zero. The alloying elemental atoms tend to randomly distribute in entropy solid solution. In contrast to an entropy solid solution, a negative enthalpy solid solution is used to describe a solid solution structure in which the chemical interactions among distinctive elements cannot be ignored; therefore, the random distribution of the constituent element atoms in the entropy solid solution is disrupted [16]. Chemical affinity-biased atomic structures ($\Delta H_{mix}^{A - B}{\mathrm{\ < \ 0\ kJ/mol}}$) are formed at different levels and include biased atomic pairs, chemical fluctuations and LCO; these are the principal features of negative enthalpy solid solutions. In general, a negative enthalpy solid solution can further evolve into a negative enthalpy alloy, highlighting the roles of negative enthalpic constituents in solid solutions, including *in situ* solid solutions with nanocomposites of intermetallic compounds and metallic glass. The negative enthalpies are indicators of the inner energies of a solid. The negative enthalpy alloy concept and route involve the use of quantified negative enthalpy values as a guide for designing metals and alloys to approach the synergy of strength and ductility, as shown in Fig. 1.

### Definition of negative enthalpy alloy

In comparison to negative enthalpy solid solutions, to be more general, negative enthalpy alloys composed of negative enthalpic crystalline or non-crystalline structures, which can be composite structures formed by negative enthalpic solid solutions with intermetallic compound alloys and/or metallic glass components, are considered negative enthalpy alloys.

## Supplementary Material

nwae026_Supplemental_File
