# Supplementary material for: Negative enthalpy alloys and local chemical ordering: a concept and route leading to synergy of strength and ductility
Source: Natl Sci Rev. 2024 Jan 18;11(4):nwae026. doi: 10.1093/nsr/nwae026 (PMC10890820; doi:10.1093/nsr/nwae026)
Supplement: nwae026_Supplemental_File [file nwae026_supplemental_file.pdf]

Supplementary materials

Negative enthalpy alloys and local chemical ordering: a concept and route leading to synergy of strength and ductility

Zibing An[1], Tao Yang[2], Caijuan Shi[3], Shengcheng Mao[1], Lihua Wang[1], Ang Li[1], Wei Li[1], Xianmeng Xue[1], Ming Sun[1], Yifan Bai[1], Yapeng He[1], Fuzeng Ren[4], Zhouguang Lu[4], Ming Yan[4], Yang Ren[5], Chain-Tsuan Liu[2], Ze Zhang[1, 6] and Xiaodong Han[1, 4*]

[1]Beijing Key Lab of Microstructure and Property of Advanced Materials, College of Materials Science & Engineering, Beijing University of Technology, Beijing 100124, China
[2]Department of Materials Science and Engineering, City University of Hong Kong, Hong Kong, China
[3]Key Laboratory of Partial Acceleration Physics & Technology, Institute of High Energy Physics, Chinese Academy of Sciences, Beijing, 100049, China
[4]Department of Materials Science & Engineering, Southern University of Science and Technology, Shenzhen 518055, China
[5]Department of Physics, City University of Hong Kong, Hong Kong, China
[6]State Key Laboratory of Silicon Materials, Department of Materials Science and Engineering, Zhejiang University, Hangzhou 310058, China

***Corresponding author**. E-mail: xdhan@bjut.edu.cn

Contents

The calculation of the mixing enthalpy……………………………………………………..…2
Material preparation ……………………..……………………………………………...…3
Microstructural and elemental characterization………..…………………………………….......…3
Mechanical property measurements…………………………………………………………...3
In situ synchrotron high-energy XRD tests……………………………………………………3
The calculation of dislocation density…………………………………………………………4
Supplementary Figure 1. XRD patterns..5
Supplementary Figure 2. Microstructure and chemical distribution 6
Supplementary Figure 3. Chemical distribution in the homogenized…………………………7
Supplementary Figure 4. TEM image and corresponding ……………………………..……..8
Supplementary Figure 5. Microstructure of $HNTTA_{18}$ alloy……………………………..…..…..9
Supplementary Figure 6. Microstructure of $HNTTV_5$ and $HNTTV_{10}$ alloys……………………….…10
Supplementary Figure 7. In situ synchrotron XRD spectrum……………………………..……11
Supplementary Table 1 Composition of $HNTTA_x$ (x=0, 5, 10, 12, 15) alloys……………………..…12
Supplementary Table 2 Mixing enthalpy ΔH_{mix} (kJ/mol) among the elements……………………..…13
Extended Alloys………………………………………………………………………………14

The calculation of the mixing enthalpy

In an HEA system, the mixing enthalpy can be calculated by [1-3]:

$$\Delta H_{mix} = 4\sum_{i<j} c_i c_j \Delta H_{ij} \quad \text{(S1)}$$

where c_i and c_j are the mole fractions of constituents i and j. ΔH_{ij} is the mixing enthalpy of constituents i and j in their binary liquid system [4], which was used for alloy composition design.

Material preparation

Melts with $(HfNbTiTa)_{100}Al_0$, $(HfNbTiTa)_{95}Al_5$, $(HfNbTiTa)_{90}Al_{10}$, $(HfNbTiTa)_{88}Al_{12}$, and $(HfNbTiTa)_{85}Al_{15}$ (at.%) nominal compositions were prepared via arc melting of a mixture of pure metals (99.95 at.%) in a Ti-gettered high-purity argon atmosphere. Prior to the arc melting process, pure Al metal particles were placed at the bottom of the copper crucible due to their low melting temperature. The particles were remelted at least ten times to ensure the initial chemical homogeneity in the melt. Moreover, the samples were turned over upon each remelting procedure. Finally, ingots were obtained by drop casting of the melt into a water-cooled copper mold (60 mm × 12 mm × 5 mm). The plates were then wrapped in the Ta foil, sealed in quartz tubes filled with pure Ar and sponge Ti atmospheres, and homogenized at 1300 ℃ for 2 h. The homogenized $(HfNbTiTa)_{100}Al_0$, $(HfNbTiTa)_{95}Al_5$, $(HfNbTiTa)_{90}Al_{10}$, and $(HfNbTiTa)_{88}Al_{12}$ plates were then cold-rolled to achieve a ~70% thickness reduction to 1.5 mm, then annealed at 1100 ℃ for 5 min under the Ar protective environment for recrystallization, and finally water-quenched to the ambient temperature. After that, the samples were aged at 400 ℃ for 120 h under the Ar atmosphere (hereafter referred to as aged alloys). Because of the brittleness, the $(HfNbTiTa)_{85}Al_{15}$ alloy was exposed to only homogenization and annealing (hereafter also referred to as the aged alloy).

Microstructural and elemental characterization

X-ray diffraction (XRD) spectra were obtained with a Cu-Kα radiation source and scanned in the 2θ range of 20–100° at a speed of 0.01° s^{-1}. The microstructure and morphology at the microscale were characterized using an FEI-Quanta 650 field-emission scanning electron microscope equipped with a TSL-EBSD system. The microstructure at the nanoscale and atomic scale of the specimens was characterized using high-resolution TEM with an aberration-corrected FEI Titan-Themis instrument and a double aberration-corrected FEI Titan-Themis-Z instrument at 300 kV. High-angle annular dark-field scanning transmission electron microscopy (STEM-HAADF) was performed with an FEI Titan-Themis-Z instrument containing a superXG2 EDS addon to analyze the atomic-resolution structure and chemical distribution.

Mechanical property measurements

Room-temperature tensile properties were measured using an INSTRON-5966 universal tensile testing machine at an initial strain rate of 5×10^{-4} s^{-1}. Dog bone-shaped tensile samples with a cross-section of 1.0×2.5 mm^2 and a gauge length of 20 mm were cut via electrical discharge machining. The strain was measured by an extensometer.

In situ synchrotron high-energy XRD tests

High-energy X-rays with an energy of 60.05 keV, a wavelength of 0.02065 nm and a beam size of 0.7 mm × 0.7 mm were used to obtain two-dimensional diffraction patterns in transmission geometry with a Perkin-Elmer large-area detector. A Gatan microtension tester equipped with a home-built tensile jig was used for in situ tensile testing. The *in situ* tensile tests were performed with a strain rate of 1×10^{-3} s^{-1}.

The calculation of dislocation density

The schematic of the *in situ* HR-XRD tensile test is shown in the inset of Fig. 6a. The loading direction (LD) and transverse direction (TD) correspond to azimuth angles of 0 and 90 degrees, respectively. The 2-D diffraction patterns were converted into one-dimensional (1-D) HE-XRD diffraction spectra by caking and integrating over a ± 5-degree range along specified azimuth angles (inset Fig. 6a). Diffraction peaks were used to determine the dislocation density at different strains according to the William-Hall method [5]. The broadening of diffraction reflection is mainly attributed to the average crystallite size (D) broadening and strain (ε) broadening.

$$B\cos\theta_{hkl} = K\lambda/D + \varepsilon\sin\theta_{hkl} \quad \text{(S2)}$$

Where B is the XRD peak broadening (full width at half maxima), K is a constant 1, λ is the wavelength of the diffraction, D is the average crystallite size, and θ_{hkl} is the Bragg angle of the diffraction peak used for the calculation. The values of D and ε at every applied strain can be calculated by the intercept and slope from the linearly fitted lines of the $B\cos\theta_{hkl}$ as a function of $\sin\theta_{hkl}$.

The dislocation density (ρ) can be determined by the following equation:

$$\rho = 3.46\varepsilon/Db \quad \text{(S3)}$$

where, b is the Burgers vector of dislocation. For the BCC structured alloy, $b=a/2\langle 111\rangle$.

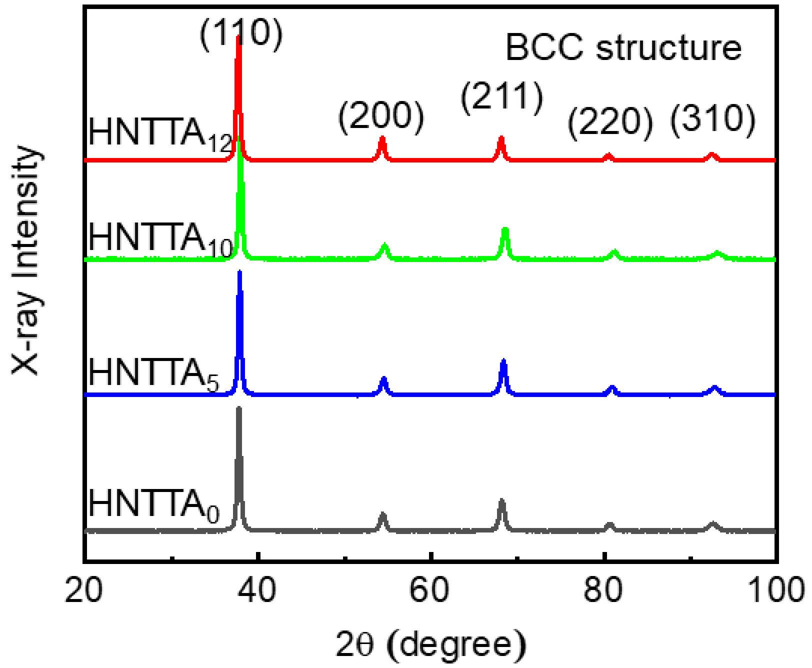

Supplementary Figure 1. XRD patterns of $HNTTA_0$, $HNTTA_5$, $HNTTA_{10}$, and $HNTTA_{12}$ alloys, all of the peaks indexed for a BCC structure.

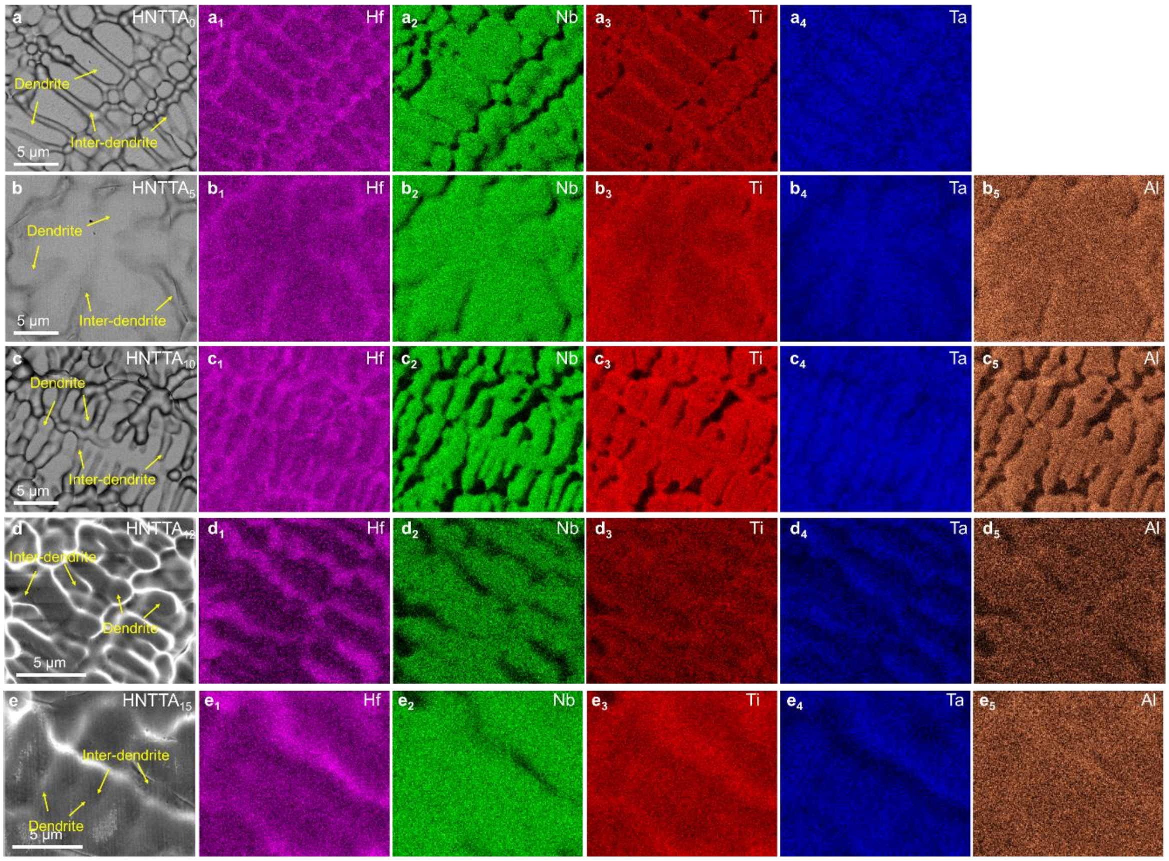

Supplementary Figure 2. Microstructure and chemical distribution of as-cast HNTTA$_x$ (x=0, 5, 10, 12, 15) alloys. a-e The SEM images of the as-cast HNTTA$_0$, HNTTA$_5$, HNTTA$_{10}$, HNTTA$_{12}$, and HNTTA$_{15}$ alloys, showing dendritic microstructure. **a$_1$-a$_4$** Corresponding EDS map of HNTTA$_0$ alloy. **b$_1$-b$_5$** Corresponding EDS map of HNTTA$_5$ alloy. **c$_1$-c$_5$** Corresponding EDS map of HNTTA$_{10}$ alloy. **d$_1$-d$_5$** Corresponding EDS map of HNTTA$_{12}$ alloy. **e$_1$-e$_5$** Corresponding EDS map of HNTTA$_{15}$ alloy.

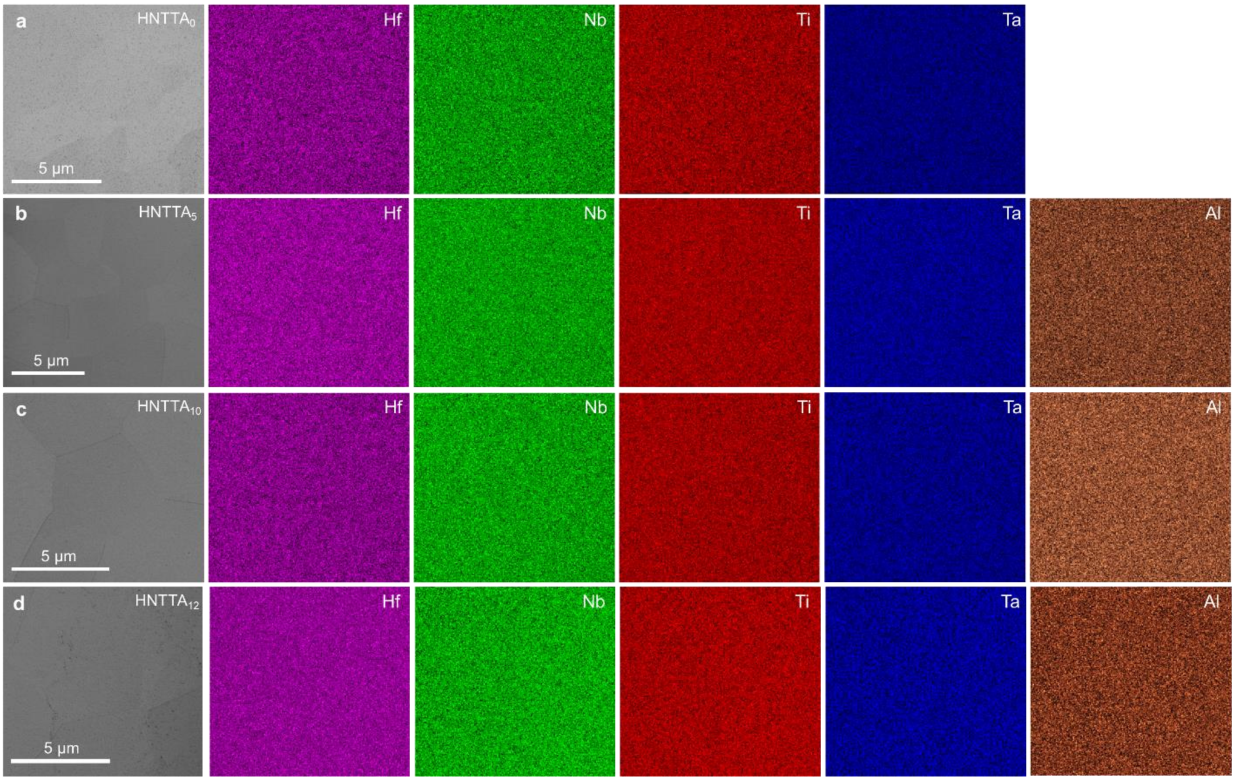

Supplementary Figure 3. Chemical distribution in the aged HNTTA$_x$ (x=0, 5, 10, 12) alloys. a SEM image and the corresponding EDS map of HNTTA$_0$ alloy. **b** SEM image and the corresponding EDS map of HNTTA$_5$ alloy. **c** SEM image and the corresponding EDS map of HNTTA$_{10}$ alloy. **d** SEM image and the corresponding EDS map of HNTTA$_{12}$ alloy.

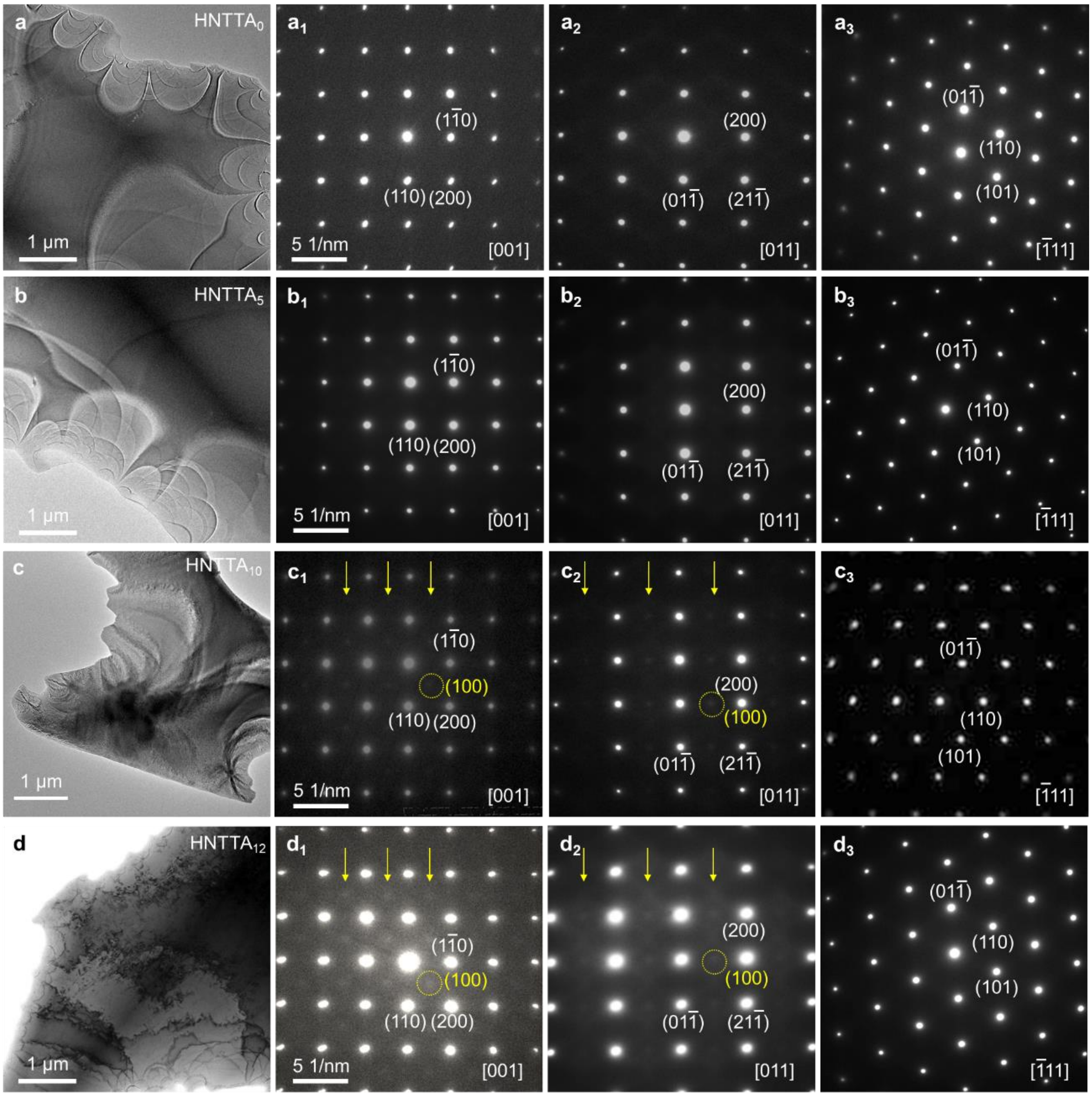

Supplementary Figure 4. TEM image and corresponding SAED pattern of aged $HNTTA_x$ alloys. **a** Bright field TEM image of $HNTTA_0$ alloy. **a_1-a_3** SAED patterns under [001], [011], and [-111] zone axis, respectively. **b** Bright field TEM image of $HNTTA_5$ alloy. **b_1-b_3** SAED patterns under [001], [011], and [-111] zone axis, respectively. **c** Bright field TEM image of $HNTTA_{10}$ alloy. **c_1-c_3** SAED patterns under [001], [011], and [-111] zone axis, respectively. **d** Bright field TEM image of $HNTTA_{12}$ alloy. **d_1-d_3** SAED patterns under [001], [011], and [-111] zone axis, respectively.

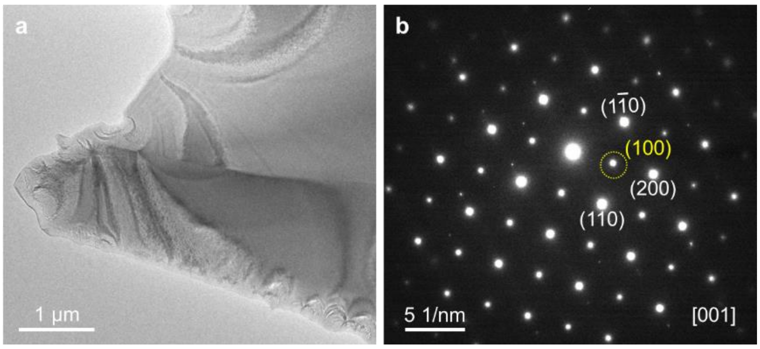

Supplementary Figure 5. Microstructure of $HNTTA_{18}$ alloy. a Bright field TEM image of $HNTTA_{18}$ alloy. **b** SAED patterns under [001] zone axis, showing B2 structure.

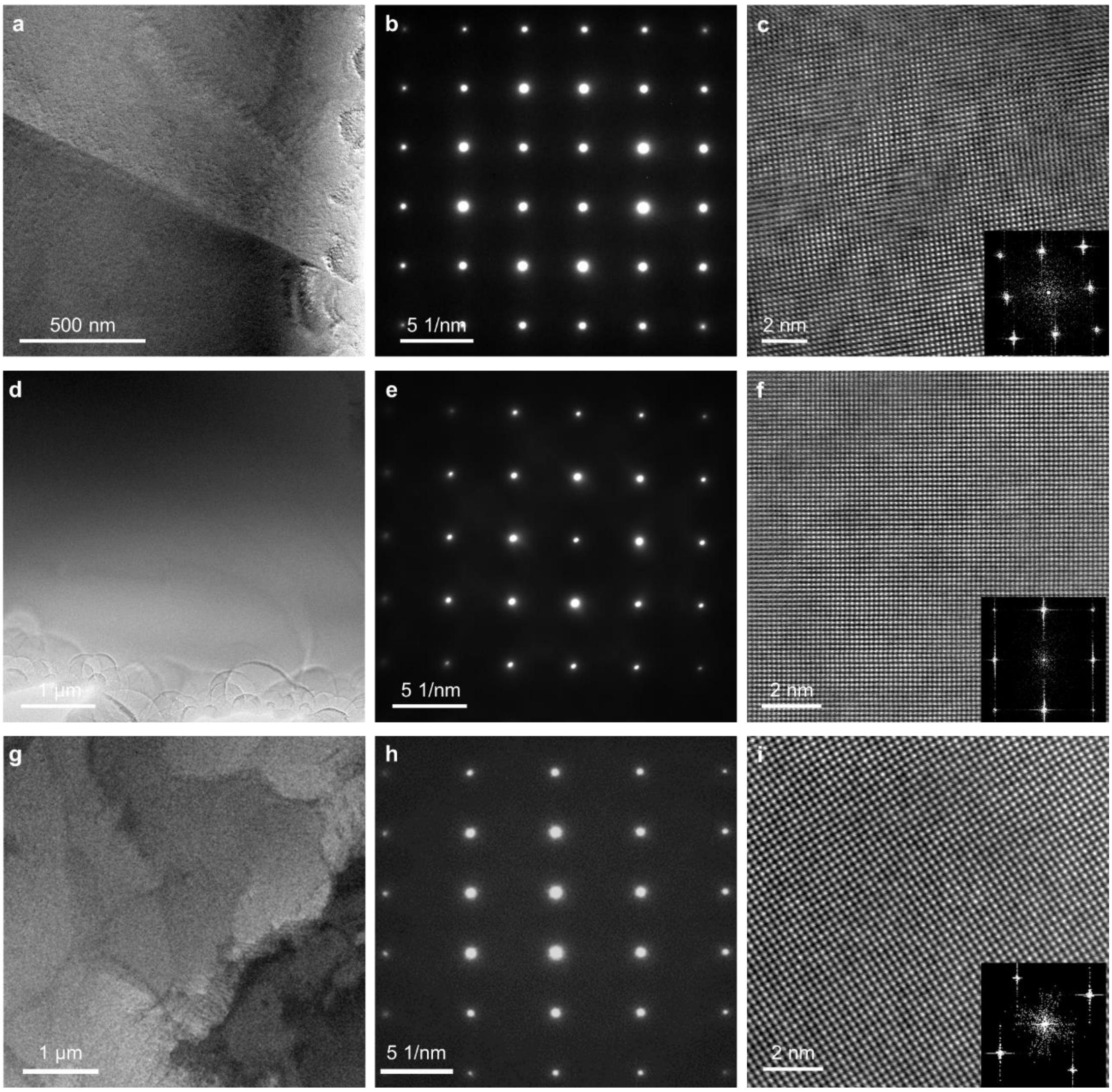

Supplementary Figure 6. Microstructure of $HNTTV_5$, $HNTTV_{10}$, and $HNTTV_{12}$ alloys. a Bright field TEM image of $HNTTV_5$ alloy. **b** SAED patterns under [001] zone axis. **c** Atomic resolution HAADF-STEM image. Inset shows the FFT. **d** Bright field TEM image of $HNTTV_{10}$ alloy. **e** SAED patterns under [001] zone axis. **f** Atomic resolution HAADF-STEM image. Inset shows the FFT. **g** Bright field TEM image of $HNTTV_{12}$ alloy. **h** SAED patterns under [011] zone axis. **i** Atomic resolution HAADF-STEM image. Inset shows the FFT.

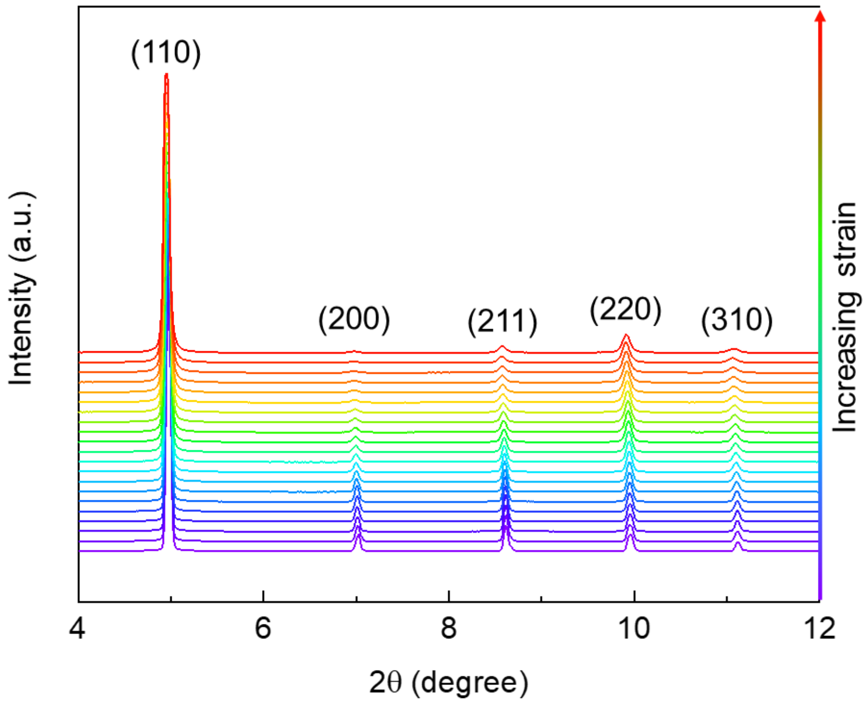

Supplementary Figure 7. In situ synchrotron XRD spectrum in varying crystal planes with increase tensile strain, confirming the BCC structure with increased micro-strain.

Supplementary Table 1 Composition of $HNTTA_x$ (x=0, 5, 10, 12, 15) alloys measured by SEM-EDS

Alloys	Elements (at.%)	Hf	Nb	Ti	Ta	Al
$HNTTA_0$	Nominal	25	25	25	25	/
	Dendrite	21.89	27.98	21.56	28.57	/
	Inter-dendrite	28.56	22.11	29.18	20.15	/
	Average	25.08	26.12	24.85	23.95	/
$HNTTA_5$	Nominal	23.75	23.75	23.75	23.75	5
	Dendrite	20.23	29.85	21.2	25.51	3.21
	Inter-dendrite	27.15	22.23	26.89	17.14	6.59
	Average	24.08	23.82	24.46	21.75	4.89
$HNTTA_{10}$	Nominal	22.5	22.5	22.5	22.5	10
	Dendrite	20.01	26.12	19.5	25.81	8.56
	Inter-dendrite	25.98	19.58	26.31	16.9	11.23
	Average	23.61	23.01	22.35	21.61	9.42
$HNTTA_{12}$	Nominal	22	22	22	22	12
	Dendrite	19.50	27.21	17.54	24.20	11.55
	Inter-dendrite	26.21	19.75	25.73	17.20	11.11
	Average	21.80	22.50	21.12	20.89	13.69
$HNTTA_{15}$	Nominal	21.25	21.25	21.25	21.25	15
	Dendrite	18.70	25.52	17.46	22.71	15.61
	Inter-dendrite	25.83	20.02	26.12	16.97	11.06
	Average	21.02	21.96	21.34	20.98	14.70

Supplementary Table 2 Mixing enthalpy ΔH_{mix} (kJ/mol) among the elements that are usually used in ductile BCC refractory HEAs [4]

	Ti	Zr	Hf	V	Nb	Ta	Al	Ru	Re
Ti		0	0	-2	2	1	-30	-42	-25
Zr			0	-4	4	3	-44	-59	-35
Hf				-2	4	3	-39	-52	-30
V					-1	-1	-16	-25	-13
Nb						0	-18	-41	-26
Ta							-19	-39	-24
Al								-21	-9
Ru									-1

Extended Alloys

Designing of local chemical ordering (LCO) structure via negative mixing enthalpy is supposed to have broad applicability in BCC refractory high-entropy alloys (RHEAs). That is because the mixing enthalpy of each pair of constituents (X-X) has a similar value in HEAs, e.g., Zr-Hf-Nb-Ta-Ti-V family BCC RHEAs [6,7] (Table S2). Negative mixing enthalpy alloys can be achieved by adding of other elements (M), e.g., Al/Ru/Re in BCC RHEAs, with significantly reduced M-X enthalpy[1]. Thus, LCO heterogeneous structure and superior strength−ductility are expected. Another two examples are as follows:

1. $Ti_{50}Zr_{20}(VNb)_{30-x}Al_x$ alloy

We fabricated novel $Ti_{50}Zr_{20}(VNb)_{30}$, $Ti_{50}Zr_{20}(VNb)_{25}Al_5$, and $Ti_{50}Zr_{20}(VNb)_{20}Al_{10}$ alloys by arc melting. The mixing enthalpy of the alloying elements is shown in Table S2. The cast alloys were followed by homogenization at 1300 °C for 2h, and then cold-rolling (~60%) and annealed at 1100 °C for 5 min, as well as aged at 400°C for 120h (hereafter named as aged alloys). The mixing enthalpies of $Ti_{50}Zr_{20}(VNb)_{30}$, $Ti_{50}Zr_{20}(VNb)_{25}Al_5$, and $Ti_{50}Zr_{20}(VNb)_{20}Al_{10}$ were determined to be -0.1 kJ/mol, -5.7 kJ/mol, and -10.9 kJ/mol, respectively. All these alloys are in a BCC solid solution structure without a second phase (Fig. S8). Fig. S9a shows BF-TEM image of the $Ti_{50}Zr_{20}(VNb)_{30}$ alloy, revealing a single-phase structure, which is consist with the XRD measurement. Fig. S9b show SAED patterns under the zone axis of [001], which is fully indexed to the BCC structure without other sign of diffraction information from any superlattice structure. Fig. S9c shows the BF-TEM image of $Ti_{50}Zr_{20}(VNb)_{25}Al_5$ alloy. Similar, the corresponding SAED patterns under the zone axis of [001] is shown in Fig. S9d. This observation also suggests that a single-phase BCC structure of $Ti_{50}Zr_{20}(VNb)_{25}Al_5$ alloy. Fig. S9e shows the BF-TEM image of $Ti_{50}Zr_{20}(VNb)_{20}Al_{10}$ alloy. Apart from a set of sharp diffraction spots from the BCC base, the extra superlattice reflections appear in the form of many weak spots in intensity under the [001] z.a. (Fig. S9f), locating at the 1/2[200] positions of BCC spots, as indicated by arrows (one is circled in yellow), indicating the LCO structure. Fig. S9g shows the atomic resolution HAADF-STEM image. Inset shows the FFT, revealing the extra superlattice reflection (blue circle), confirming the LCO structure. The LCO entities were revealed by the corresponding IFFT image by using extra reflections (1/2{200} spots), as shown in Fig. S9h. Compared with $Ti_{50}Zr_{20}(VNb)_{30}$, $Ti_{50}Zr_{20}(VNb)_{25}Al_5$ alloys, featuring a similar state with different mixing enthalpy, $Ti_{50}Zr_{20}(VNb)_{20}Al_{10}$ alloy shows LCO structure, which further demonstrate that negative mixing enthalpy is the genic for the formation of LCO structure. The $Ti_{50}Zr_{20}(VNb)_{20}Al_{10}$ alloy shows an ultrahigh yield strength, reaching up to 1010 MPa (Fig. S10), which is approximately 318 MPa higher than that of $Ti_{50}Zr_{20}(VNb)_{30}$ alloy, showing a negative mixing enthalpy strengthening with coefficient ($|K|$) of ~30 MPa/(kJ/mol), in line with the results of $HNTTA_x$ alloy. The $Ti_{50}Zr_{20}(VNb)_{20}Al_{10}$ alloy also exhibits large uniform tensile strain of ~22%. By contrast, the $Ti_{50}Zr_{20}(VNb)_{30}$ alloy shows necking after yield as soon as possible, indicating no uniform tensile ductility.

1. $Ti_{50}Zr_{20}(VNb)_{30-x}Ru_x$ alloy

We fabricated $Ti_{50}Zr_{20}(VNb)_{29}Ru_1$, $Ti_{50}Zr_{20}(VNb)_{27}Ru_3$, and $Ti_{50}Zr_{20}(VNb)_{25}Ru_5$ alloys by arc melting. The mixing enthalpy of the alloying elements is shown in Table S2. The cast alloys were followed by homogenization at 1300 °C for 2h, and then cold-rolling (~60%) and annealed at 1100 °C for 5 min, as well as aged at 400°C for 120h (hereafter named as aged alloys). The mixing enthalpies of $Ti_{50}Zr_{20}(VNb)_{29}Ru_1$, $Ti_{50}Zr_{20}(VNb)_{27}Ru_3$, and $Ti_{50}Zr_{20}(VNb)_{25}Ru_5$ were determined to be -0.1 kJ/mol, -5.1 kJ/mol, and -8.4 kJ/mol, respectively. All these alloys are in a BCC solid solution structure without a second phase (Fig. S11). Fig. S12a shows BF-TEM image of the $Ti_{50}Zr_{20}(VNb)_{29}Ru_1$ alloy, revealing a single-phase structure. Corresponding SAED patterns under the zone axis of [001] is shown in Fig. 12b, indicating an ideal BCC solid solution structure. Fig. S12c shows the BF-TEM image of $Ti_{50}Zr_{20}(VNb)_{27}Ru_3$ alloy. The corresponding SAED patterns under the [011] zone axis is shown in Fig. S12d, suggesting an ideal BCC solid solution structure. Fig. S12e shows the BF-TEM image of $Ti_{50}Zr_{20}(VNb)_{25}Ru_5$ alloy. Apart from a set of sharp diffraction spots from the BCC base, the extra superlattice reflections appear in the form of many weak spots in intensity under the [001] z.a. (Fig. S12f), locating at the 1/2[200] positions of BCC spots, as indicated by arrows (one is circled in yellow). This result provides an indication that the negative mixing enthalpy $Ti_{50}Zr_{20}(VNb)_{25}Ru_5$ alloy show LCO structure, similar with the $HNTTA_{10}$ and $Ti_{50}Zr_{20}(VNb)_{20}Al_{10}$ alloys. Fig. S12g shows the atomic resolution HAADF-STEM image. Inset shows the FFT, revealing the extra superlattice reflection (blue circle), confirming the LCO structure. The LCO entities were revealed by the corresponding IFFT image by using extra reflections (1/2{200} spots), as shown in Fig. S12h. Though, no Al element addition, the Ru elements can also decrease the mixing enthalpy of the system, exhibiting LCO structure in the $Ti_{50}Zr_{20}(VNb)_{25}Ru_5$ alloy. Thus, it is can be demonstrated that negative mixing enthalpy is the genic for the formation of LCO structure. It is anticipated that the $Ti_{50}Zr_{20}(VNb)_{25}Ru_5$ alloy shows an ultrahigh yield strength, reaching up to 1230 MPa (Fig. S13), which is approximately 110 MPa higher than that of $Ti_{50}Zr_{20}(VNb)_{29}Ru_1$ alloy, showing a negative mixing enthalpy strengthening with coefficient ($|K|$) of ~32 MPa/(kJ/mol), in line with the results of $HNTTA_x$, and $Ti_{50}Zr_{20}(VNb)_{30-x}Al_x$ alloys. The $Ti_{50}Zr_{20}(VNb)_{25}Ru_5$ alloy exhibits large uniform tensile strain of ~21.5%. By contrast, the $Ti_{50}Zr_{20}(VNb)_{29}Ru_1$ alloy shows an uniform tensile strain of ~15.0%.

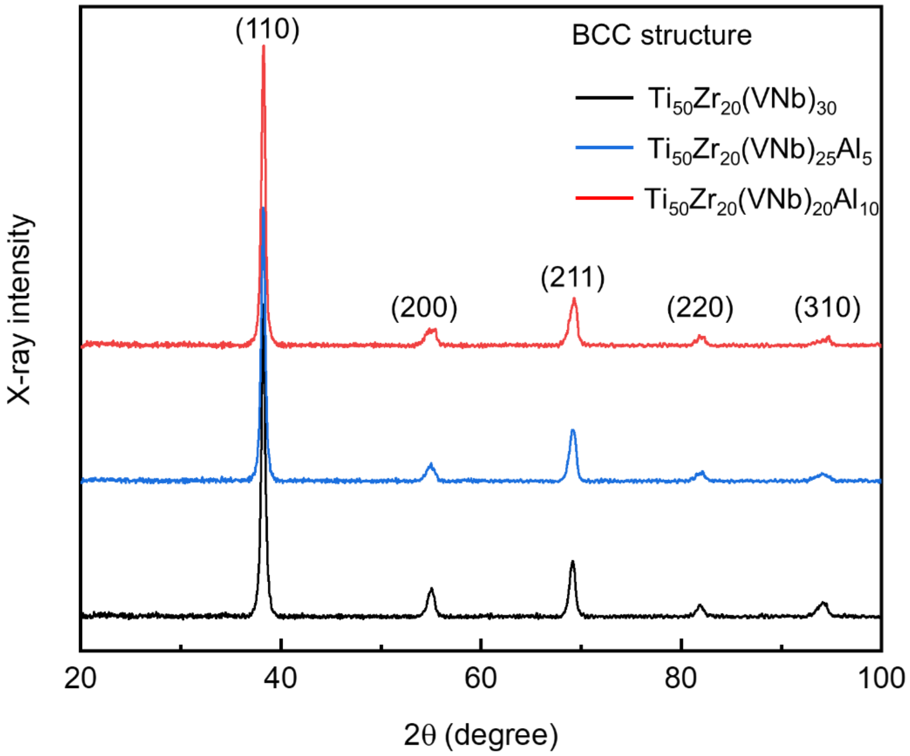

Supplementary Figure 8. XRD patterns of aged $Ti_{50}Zr_{20}(VNb)_{30}$, $Ti_{50}Zr_{20}(VNb)_{25}Al_5$, and $Ti_{50}Zr_{20}(VNb)_{20}Al_{10}$ alloys, all of the peaks indexed for a BCC structure.

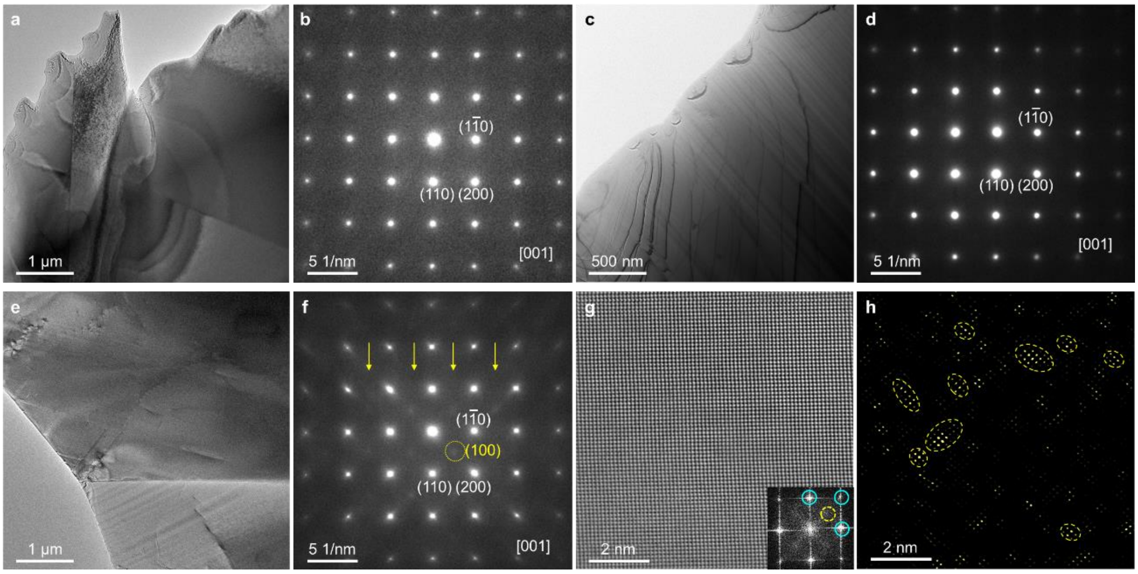

Supplementary Figure 9. Microstructure of aged $Ti_{50}Zr_{20}(VNb)_{30}$, $Ti_{50}Zr_{20}(VNb)_{25}Al_5$, and $Ti_{50}Zr_{20}(VNb)_{20}Al_{10}$ alloys. a BF-TEM image of aged $Ti_{50}Zr_{20}(VNb)_{30}$ alloy. **b** Corresponding SAED pattern under [001] z.a. **c** BF-TEM image of aged $Ti_{50}Zr_{20}(VNb)_{25}Al_5$ alloy. **d** Corresponding SAED pattern under [001] z.a. **e** BF-TEM image of aged $Ti_{50}Zr_{20}(VNb)_{20}Al_{10}$ alloy. **f** Corresponding SAED pattern under [001] z.a. The yellow arrows/circle shows the extra reflection. **g** Atomic resolution HAADF-STEM image. Inset shows the FFT. Yellow circle: extra reflection. Blue circle: BCC lattice reflection. **h** IFFT image, revealing LCO structure.

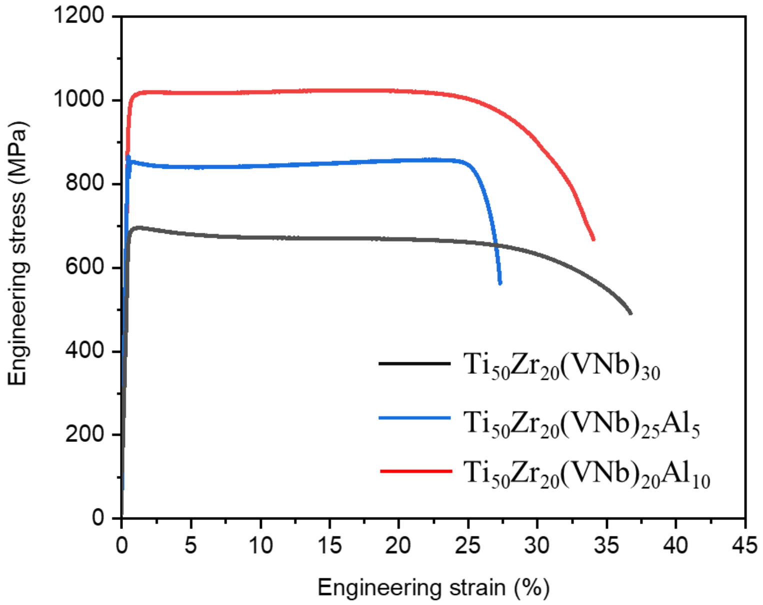

Supplementary Figure 10. Engineering stress–strain curves of aged $Ti_{50}Zr_{20}(VNb)_{30}$, $Ti_{50}Zr_{20}(VNb)_{25}Al_5$, and $Ti_{50}Zr_{20}(VNb)_{20}Al_{10}$ alloys under tensile deformation.

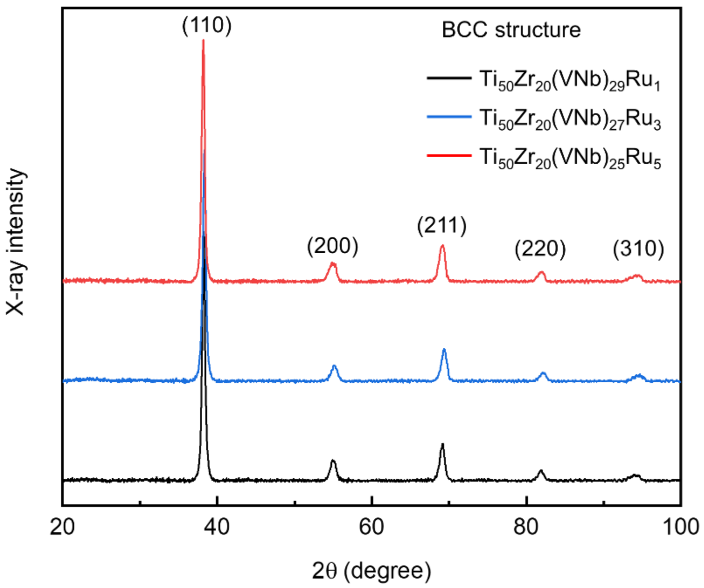

Supplementary Figure 11. XRD patterns of aged $Ti_{50}Zr_{20}(VNb)_{29}Ru_1$, $Ti_{50}Zr_{20}(VNb)_{27}Ru_3$, and $Ti_{50}Zr_{20}(VNb)_{25}Ru_5$ alloys, all of the peaks indexed for a BCC structure.

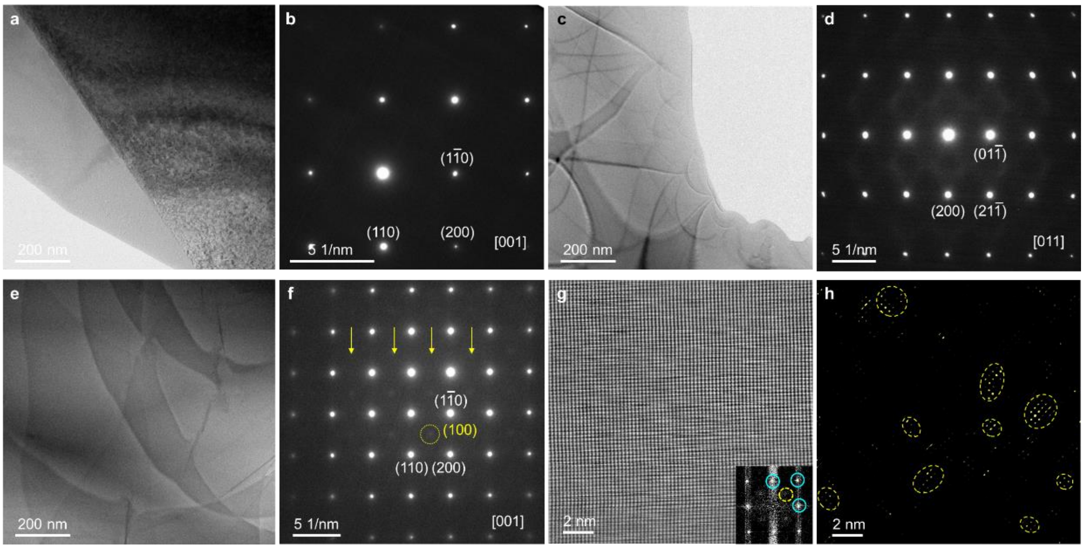

Supplementary Figure 12. Microstructure of aged $Ti_{50}Zr_{20}(VNb)_{29}Ru_1$, $Ti_{50}Zr_{20}(VNb)_{27}Ru_3$, and $Ti_{50}Zr_{20}(VNb)_{25}Ru_5$ alloys. **a** BF-TEM image of aged $Ti_{50}Zr_{20}(VNb)_{29}Ru_1$ alloy. **b** Corresponding SAED pattern under [001] z.a. **c** BF-TEM image of aged $Ti_{50}Zr_{20}(VNb)_{27}Ru_3$ alloy. **d** Corresponding SAED pattern under [011] z.a. **e** BF-TEM image of aged $Ti_{50}Zr_{20}(VNb)_{25}Ru_5$ alloy. **f** Corresponding SAED pattern under [001] z.a. The yellow arrows/circle shows the extra reflection. **g** Atomic resolution HAADF-STEM image. Inset shows the FFT. Yellow circle: extra reflection. Blue circle: BCC lattice reflection. **h** IFFT image, revealing LCO structure.

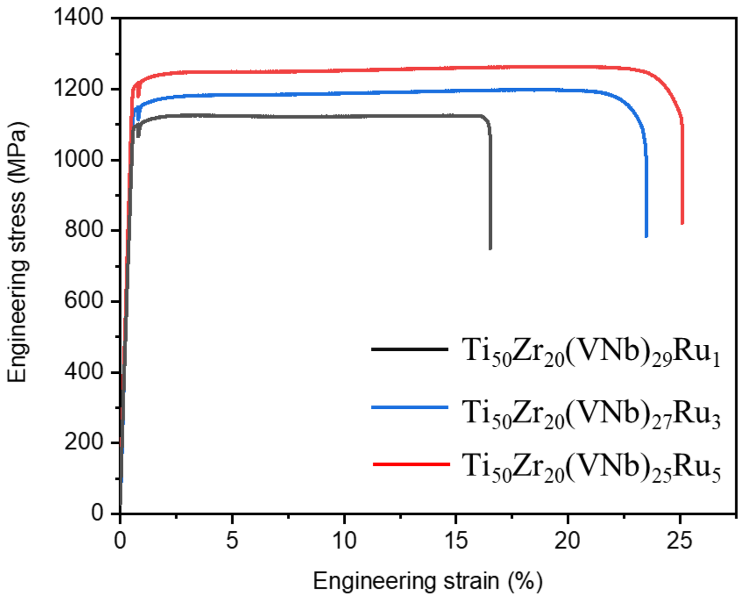

Supplementary Figure 13. Engineering stress–strain curves of aged $Ti_{50}Zr_{20}(VNb)_{29}Ru_1$, $Ti_{50}Zr_{20}(VNb)_{27}Ru_3$, and $Ti_{50}Zr_{20}(VNb)_{25}Ru_5$ alloys under tensile deformation.

REFFERENCES

1. Zhang Y *et al*. Solid-solution phase formation rules for multi-component alloys. Adv Eng Mater 2009; **10**(6): 534-8.
2. Takeuchi A *et al*. Quantitative evaluation of critical cooling rate for metallic glasses. Mater. Sci. Eng. A 2001; **446**: 304–6.
3. Miedema *et al*. cohesion in alloys – fundamentals of a semi-empirical model. Phys. B 1980; **100**: 1.
4. Takeuchi A *et al*. Classification of bulk metallic glasses by atomic size difference, heat of mixing and period of constituent elements and its application to characterization of the main alloying element. Mater Trans 2005; **46**(12): 2817-29.
5. Xu N *et al*. Micromechanical behaviors of $Fe_{20}Co_{30}Cr_{25}Ni_{25}$ high entropy alloys with partially and completely recrystallized microstructures investigated by in-situ high-energy x-ray diffraction. Metal Mater Trans A 2021; **52**(9): 3674-83.
6. Qiao, D *et al.* A Novel Series of Refractory High-Entropy Alloys $Ti_2ZrHf_{0.5}VNb_x$ with High Specific Yield Strength and Good Ductility. Acta Metall Sin 2019; **32**; 925-31.
7. Wu, Z *et al*. Temperature dependence of the mechanical properties of equiatomic solid solution alloys with face-centered cubic crystal structures. Acta Mater 2014; **81**: 428-41.
